# Supplementary material for: Risk Stratification in Hypertrophic Cardiomyopathy. Insights from Genetic Analysis and Cardiopulmonary Exercise Testing
Source: J Clin Med. 2020 May 28;9(6):1636. doi: 10.3390/jcm9061636 (PMC7356142; doi:10.3390/jcm9061636)
Supplement: Supplementary file 1 [file jcm-09-01636-s001.zip › Genetics Vs CPET in HCM/Genetics versus CPET in HCM.docx]

**CARDIOPULMONARY EXERCISE TESTING OVER GENETIC TESTING ANALYSIS**

**IN RISK STRATIFICATION OF HYPERTROPHIC CARDIOMYOPATHY PATIENTS**

**Running Title:** CPET and Genetics in HCM

**Authors:**

Damiano Magrì^1^, MD, PhD, Vittoria Mastromarino^1,2^, MD, PhD, Giovanna Gallo^1^, MD, Elisabetta Zachara^3^, MD, Federica Re^3^, MD, Piergiuseppe Agostoni^4,5^, MD, PhD, Dario Giordano^1^, MD, Speranza Rubattu^1,6^, MD, Maurizio Forte^6^, PhD, Maria Cotugno^6^, BS, Maria Rosaria Torrisi^7^, PhD, Simona Petrucci^1,7^, MD, PhD, Aldo Germani ^1,7^, BS, Camilla Savio^7^, BS, Antonello Maruotti^8,9,10^, PhD, Massimo Volpe^1,6^, MD, Camillo Autore^1^, MD, Maria Piane^7^, PhD, and Beatrice Musumeci^1^, MD, PhD.

**Affiliations:**

1 Dpt Clinical and Molecular Medicine, Sapienza University, Rome – Italy

2 Unit of Pediatric Cardiology and Cardiac Surgery, Sant’Orsola Hospital – Bologna – Italy

3 Cardiac Arrhythmia Center and Cardiomyopathies Unit, San Camillo - Forlanini Hospital, Rome – Italy

4 Centro Cardiologico Monzino, IRCCS, Milano – Italy

5 Dpt of Clinical Sciences and Community Health, University of Milan, Milan – Italy

6 IRCCS - Neuromed, Pozzilli (IS) – Italy

7 UOC Medical Genetics and Advanced Cell Diagnostics, S. Andrea University Hospital, Rome – Italy

8 Dpt of Scienze economiche, politiche e delle lingue moderne - Libera Università SS Maria Assunta, Rome – Italy

9 Department of Mathematics, University of Bergen - Norway

10 School of Computing, University of Portsmouth - UK

**Correspondence to:**

Magrì Damiano, MD, PhD

Department of Clinical and Molecular Medicine, “Sapienza” University - Rome - Italy

Cardiology Unit, Sant’Andrea Hospital, Via di Grottarossa 1037 – 00189 - Rome – Italy

Email: [damiano.magri@uniroma1.it](mailto:damiano.magri@uniroma1.it)

Fax: +39 06 33775061

**ABSTRACT**

The role of genetic testing over the clinical and functional variables, including data from cardiopulmonary exercise test (CPET), in the hypertrophic cardiomyopathy (HCM) risk stratification remains unclear. A retrospective genotype-phenotype correlation was performed to analyze possible differences between patients with and without likely pathogenic/pathogenic (LP/P) variants.

A total of 371 HCM patients were screened at least for the sarcomeric genes MYBPC3 (myosin binding protein C, cardiac), MYH7 (β-myosin heavy chain, cardiac), TNNI3 (troponin I, cardiac) and TNNT2 (troponin T type 2, cardiac): 203 patients had at least a LP/P variant, 23 patients had a variant of uncertain significance (VUS) and 145 patients did not show any LP/P variant or VUS. During a median 5.4years follow-up, 51 and 14 patients reached the HF and the SCD end-point, respectively. The LP/P variant was associated with a more aggressive HCM-phenotype but, at multivariate analysis, a significant effect for the HF end-point was found for left atrial diameter (LAd), circulatory-power (peak oxygen uptake*peak systolic blood pressure, CP%) and ventilatory efficiency (C-index=0.839) while only LAd and CP% were associated to the SCD end-point (C-index=0.738).

The present study reaffirms the pivotal role of the clinical variables and, particularly of those CPET-derived, in the HCM risk stratification.

**1. INTRODUCTION**

Hypertrophic cardiomyopathy (HCM), the most common genetic heart disease, inherited with an autosomal dominant pattern, incomplete penetrance and variable expressivity, is characterised by markedly different instrumental and clinical spectra [1-3]. Accordingly, there is always great interest in investigating approaches potentially able to identify early those HCM patients at high risk of cardiovascular events both in terms of sudden cardiac death (SCD) and heart failure (HF). Indeed, albeit the SCD is a devastating but relatively rare event, the HF development and its related complications represents still an incoming concern in HCM patients [4-6].

In such a context, proper due to the its genetic nature, many researchers attempted not only a HCM genotype-phenotypes correlation but also a possible genotype-based risk stratification. Indeed, HCM is predominantly a sarcomeric disease and variants in *MYH7* and *MYBPC3* genes, encoding for the cardiac thick myofilament proteins β-myosin heavy chain and myosin binding protein-C respectively, together account about 50% of the HCM families whereas likely pathogenic/pathogenic (LP/P) variants in thin filament protein genes, such as *TNNT2*, *TNNI3* and *TPM1* encoding for cardiac troponin T, troponin I and alpha-tropomyosin account less than 10% [6-9]. Several studies have demonstrated that being carriers of sarcomeric variants might exert a negative prognostic impact on outcome, as well as to have multiple simultaneous variants, the so called “gene dosage effect” [8-13]. What remains unclear, up to now, is whether the genetic profile of the single HCM patient might provide a real significant incremental risk prediction beyond the clinical risk factors, including those derived from a maximal cardiopulmonary exercise test (CPET) [2,3]. Indeed, growing evidences suggest that a CPET assessment, combined with other clinical and instrumental variables, represents a useful tool in stratifying both the SCD and the HF-related events risk in HCM patients [14-18].

Therefore, the current multicenter retrospective study investigates a possible adjunctive role of genetic testing analysis in the HCM patients’ management over the main clinical and functional parameters. Particularly, a genotype-phenotype correlation was performed to analyze possible differences between HCM patients with and without like LP/P variants with respect to their main clinical and functional features and, mainly, their SCD and HF-related events’ rate.

**2. METHODS**

**2.1 Study Sample**

Data from a total of 665 consecutive outpatients with HCM were analyzed. All patients were part of a cohort recruited and prospectively followed in three HCM Italian centers between September 2007 and December 2019: Azienda Ospedaliera Universitaria Sant’Andrea – “Sapienza” University– Rome (n. 437); Azienda Ospedaliera San Camillo Forlanini – Rome (n. 189); Centro Cardiologico Monzino – University of Milan – Milan (n. 39). The diagnosis of HCM was based on a maximal wall thickness (MWT) ≥15 mm unexplained by abnormal loading conditions or in accordance with published criteria for the diagnosis of disease in relatives of patients with unequivocal disease [2,3]. Patients with known metabolic diseases or syndromic causes of HCM were excluded from the present study.

The study complied with the ethical standards of the Declaration of Helsinki and were reviewed and approved by the institutional ethics committees. Written informed consent was obtained from all participants. The authors from each participating centre guarantee the integrity of data from their institution and have agreed to the article as written.

**2.2 Patients Clinical and Functional Assessment**

Data were independently collected at each participating centre using uniform methodology. Each HCM patient underwent a clinical assessment, including anamnesis with pedigree analysis and New York Heart Association (NYHA) classification, 24-hours ECG Holter monitoring, transthoracic Doppler echocardiography and maximal CPET. The usual five SCD risk factors were also collected [2,3]: a) FH-SCD (history of HCM-related SCD in at least one first-degree or other relatives younger than 50 years old); b) massive left ventricular (LV) hypertrophy (maximal wall thickness, MWT, ≥ 30 mm); c) at least one run of non sustained ventricular tachycardia (NSVT) (≥ 3 consecutive ventricular beats at a rate of ≥ 120 beat per minute and < 30 seconds in duration on 24-hours ECG Holter monitoring); d) unexplained syncope judged inconsistent with neurocardiogenic origin; e) abnormal blood pressure response to exercise (ABPRE) (failure to increase systolic blood pressure, SBP, by at least 20 mm Hg from rest to peak exercise or a fall of ≥ 20 mm Hg from SBP).

The following echocardiographic measurements, obtained according to the international guidelines, [19] were considered: LV end-diastolic diameter (LVEDd, parasternal long axis), the greatest LV thickness (MWT, measured at any LV site), left atrial diameter (LAd, parasternal long axis), the highest maximal LV outflow tract gradient among those measured at rest, in orthostatic position and after Valsalva maneuver (LVOTGmax, apical four-chamber view) [20] and LV ejection fraction with Simpson’s biplane methods (LVEF, apical four-chamber view).

All CPETs were performed using an electronically braked cycle-ergometer. A personalized ramp exercise protocol was performed, aiming at a test duration of 10±2 minutes [21]. The exercise was preceded by few minutes of resting breath-by-breath gas exchange monitoring and by an unloaded warm-up. In the absence of clinical events, CPET was interrupted when patients stated that they had reached maximal effort. A 12-lead ECG, diastolic and systolic blood pressure were recorded during CPET, in order to obtain the following parameters: rest heart rate (HR), peak HR, %pHR ([peak HR / (220-age)] * 100), and ΔSBP (peak SBP – rest SBP) [22]. A breath-by-breath analysis of expiratory gases and ventilation (VE) has been performed, and peak values were obtained in the last 20 seconds of exercise. The predicted peak VO_2_ was determined by using the gender-, age-, and weight-adjusted formula. Circulatory power (CP = peak VO_2_ * SBP) was obtained considering peak VO_2_ value as percentage of predicted (CP%) [19,23]. Anaerobic threshold (AT) was measured by V-slope analysis of VO_2_ and VCO_2_, and it was confirmed by ventilator equivalents and end-tidal pressures of CO_2_ and O_2_ [24]. The end of the isocapnic buffering period was identified when VE/VCO2 increased and end-tidal pressure of CO_2_ decreased. VE/VCO_2_ slope was calculated as the slope of the linear relationship between VE and VCO_2_ from the 1st minute after the beginning of the loaded exercise and the end of the isocapnic buffering period [24].

**2.3 Genetic testing**

All patients included in the study received genetic counselling and underwent genetic testing for HCM, performed by Sanger sequencing (from 2007 to 2010) or NGS (Next generation sequencing) (from 2011 to 2019). All coding regions and boundaries of flanking introns ± 25 have been analysed for each tested gene. In this retrospective study we reported the genotype-phenotype correlation analysis only for those patients screened at least for the sarcomeric genes MYBPC3 (myosin binding protein C, cardiac) MYH7 (β-myosin heavy chain, cardiac), TNNI3 (troponin I, cardiac) and TNNT2 (troponin T type 2, cardiac). Patients with variants located in non-sarcomeric genes were also excluded.

All the identified variants have been re-evaluated based on new evidence from the scientific literature and classified according to the criteria of the American College of Medical Genetics and Genomics (ACMG) [7,25]. Only the genetic variants predicted to alter the protein and with a minor allele frequency (MAF) ≤ 0.2 % (considering the prevalence of HCM disease in the general population), were considered. For this evaluation, we used MAF data derived from GnomAD (Genome Aggregation Database https://gnomad.broadinstitute.org/). The clinical classification of variants was carried out according to the 5 classes system: benign (B), likely benign (LB), variant of uncertain clinical meaning, (VUS), likely pathogenic (LP) pathogenic (P). Genetic results were considered informative for LP or P variants, non-informative for B, LB or VUS variants. Variants are reported using the Human Genome Variation Society nomenclature guidelines (<https://varnomen.hgvs.org/>).

**2.4 Clinical Outcomes**

All patients had planned clinical reviews every 6-12 months or earlier according to the clinical status. Follow-up duration was defined as the time interval between the clinical examination and either the first event or the last visit/telephone interview in case of no events.

The HF end-point was represented by the following events: death due to HF, cardiac transplantation, progression to a stable NYHA class III-IV due to an end-stage phase with or without LVEF < 50% (hypokinetic dilated phase or restrictive phenotype evolution), severe functional deterioration leading to hospitalization for septal reduction, hospitalization due to HF symptoms or signs development. The SCD end-point was also tested, which included SCD or an equivalent event. SCD was defined as witnessed sudden death with or without documented ventricular fibrillation or death within 1 h of new symptoms or nocturnal deaths with no antecedent history of worsening symptoms. Aborted SCD during follow-up and appropriate ICD therapies (defined as intervention triggered by ventricular fibrillation or rapid ventricular tachycardia at >180 bpm) were considered equivalent to SCD in accordance with previous studies [17,18,26,27].

The causes of death, as well as the other events, were ascertained by experienced cardiologists at each centre using hospital and primary health care records, death certificates, post-mortem reports, and interviews with relatives and/or physicians. Death due to non-cardiovascular causes as well as ischemic or hemorrhagic stroke and non-fatal cerebrovascular were excluded from the survival analysis.

**2.5 Statistical Analysis**

All data are expressed as mean ± standard deviation or as absolute number (percentage). Preliminarily, an extension of the Shapiro-Wilk test of normality was performed. Categorical variables were compared with a difference between proportion tests whereas a two-sample t test was used to compare the continuous data between the two study groups (No variants and VUS Versus LP/P variants). In comparing the two populations, the variance was estimated separately for both groups and the Welch-Satterthwaite modification to the degrees of freedom was used.

We therefore focused on the distribution of the survival times by adopting the Cox proportional hazards regression model. We performed a backward selection of the predictors to be included in the model. A 5% significance level was used in the backward elimination procedure to select covariates for the final multivariate model for the combined as well as the HF end-point while, due to the low events’ number, a 15% significance level was adopted for the SCD one. To avoid the inclusion of collinear variables in the multivariate Cox analysis, we built several models in which VO_2_-derived variables, known to be collinear, were added to the prognostic model one at a time. We retain the model with the best trade-off between model complexity and model fit judged by the log-likelihood. We also performed a calibration analysis. We computed the average calibration error for both approaches and tested the observed versus average predicted probabilities for each class of risk. The Brier quadratic error score and a χ2 test of goodness of fit based on the Brier score were also checked. We did not find any clear indication of over fitting from the post hoc analysis and, consequently, in the present paper we simply reported results based on the backward elimination procedure only. Discrimination of variables included in the final multivariate model specification was performed by Harrell’s C index. Therefore, we investigated proportional hazards assumption by tests and graphical diagnostics based on scaled Schoenfeld residuals. Test of proportional hazards assumption for each covariate was obtained by correlating the corresponding set of scaled Schoenfeld residuals with the Kaplan-Meier estimate of the survival distribution. To check for the presence of influential observations, we produced a matrix of estimated changes in the regression coefficients upon deleting each observation in turn and comparing the magnitudes of the largest values to the regression coefficients.

Statistical analysis was performed using R (R Development Core Team, 2014). A p value lower than or equal to 0.05 was generally considered as statistically significant.

**3. RESULTS**

From an initial study sample of 665 consecutive HCM outpatients, a total of 294 patients (44%) were excluded because they did not undergo genetic testing (n. 197), because they were lost at follow-up (n. 39), because of the presence of non-sarcomeric variants (n. 22) or, eventually, because the genetic analysis was not performed according to the previously described inclusion criteria (n. 36). Thus, a total of 371 HCM patients were effectively enrolled and analyzed in the present study. The diagram displayed in figure 1 resumes the step-by-step procedures of the population studied.

**3.1 Genetic results**

Two hundred and three (55%) genetic tests were informative, as they detected at least a LP/P variant, whereas the percentage of patients with VUS was 6% (n. 23 patients); 39% (n. 145 patients) did not show any P/LP variant or VUS (figure 2A). Excluding the B/LB variants, 124 unique variants were identified and detailed extensively in the Supplementary files (table 1S and table 2S). These variants included 91 (73%) missense, 2 (2%) intronic, 10 (8%) frameshift, 8 (6%) splicing, 11 (9%) nonsense, 1 (1%) inframe variants (figure 1S). According to ACMG criteria, 94 variants were classified as LP/P and 30 of them as VUS. *MYBPC3* and *MYH7* resulted the most mutated genes with 88 LP/P variants (75%) (figure 2S). Twenty-four patients resulted to be carriers of multiple variants, considering those with at least one P/LP variants and other P/LP or VUS variants (table 3S). Among these, 14 patients were double heterozygous with one variant in two different genes while the remaining 10 had multiple variants in the same gene. It was not possible to determinate the phase of these variants, since segregation studies among relatives could not be performed. (figure 2B).

**3.2** **Clinical and functional characteristics**

The demographic and clinical data of the entire cohort are reported in table 1. The population mainly consisted in middle-aged predominantly male (64%) patients with a quite preserved NYHA class (NYHA I-II 94%). At the study run-in, an echocardiographic evidence of end-stage phase was present in 4%, atrial fibrillation in 3% and a septal myectomy had been performed in 11% patients. Documented cardiovascular comorbidities included systemic hypertension (27%), diabetes (6%) and coronary artery disease (4%).

Table 2 shows the comparison of clinical features between patients with and without LP/P variants. The LP/P variants group showed a younger age, both at the CPET assessment and at the diagnosis, a slightly higher prevalence of FH-SCD and ABPRE and a worse functional capacity in terms of pVO_2_, CP% and VE/VCO_2_ slope. With respect to the other clinical features, at the study run-in, the LP/P variants group had a greater prevalence of patients with end-stage phase (6% Vs 2%, p=0.011) and atrial fibrillation (3% Vs 1%, p=0.032), whereas no difference in prevalence of previous myectomy was found (5% for both groups). Concerning the documented cardiovascular comorbidities, no difference was found in coronary artery disease (3% Vs 5%), whereas the LP/P variants group showed a lower prevalence of systemic hypertension (15% Vs 31%, p<0.001) and diabetes (3% Vs 8%, p=0.007).

**3.3 End-Point Analysis**

Median follow-up was 5.4 years (25th-75th centile: 2.3 to 8.1 years) with a total of 2,271 patient-year. During the entire follow-up, a total of 129 (35%) patients experienced at least one of the pre-specified events. In patients who developed multiple events, time to the first one was used as an event time cutoff and, accordingly, a SCD or HF-related events at 5-years cumulative hazard equal to 0.369 was estimated. Patients who ended the follow-up period before the tenth year were considered censored at the time of the last clinical evaluation.

A total of 14 SCD or SCD-equivalents were analyzed. Specifically, SCD occurred in 3 patients, 4 patients experienced a resuscitated SCD, 7 patients had an appropriate ICD intervention. A total of 52 HF-related events were analyzed. Specifically, HF-related death occurred in 2 patients, 7 patients underwent cardiac transplantation, 20 patients were hospitalized due to HF signs/symptoms, 12 patients were hospitalized for septal reduction procedure due to significant HF signs/symptoms development and 11 patients evolved to end-stage or restrictive phenotype evolution. Table 3 reports the detailed Cox proportional univariate survival analysis for both the study end-points. Most of the single variables were significantly associated with the HF end-point whereas few of them to the SCD end-point. Particularly, besides a number of clinical variable, the LP/P variant presence was significantly associated to the HF but not to the SCD end-point (figure 3). Instead, at multivariate analysis, covariates showing significant effects for the primary HF-related end-point were the following: LAd, CP% and VE/VCO_2_ slope (C-index 0.839, p < 0.001) while LAd and the CP% remained independently associated to the SCD end-point (C-index 0.738).

**4. DISCUSSION**

The present multicenter retrospective study, conducted on a suitable cohort of consecutive HCM outpatients regularly followed at 3 Italian tertiary HCM centers, shows that HCM patients with LP/P sarcomeric variants tend to present a more aggressive form of disease with an earlier onset, a worse functional status and a greater risk of HF development and HF-related complications, compared to HCM patients with VUS or any variants. However, contextually, our data do not support a strict role of the genetic testing over a HCM patients’ comprehensive clinical assessment. Indeed, the LP/P variants presence does not emerge at multivariate analysis as an independent risk factor, being other instrumental variables (i.e. LAd, CP% and VE/VCO2 slope) much stronger outcome predictors.

Over the last decades a number of clinical features have been investigated in order to better understand the HCM pathophysiology as well as to identify those patients at high risk of adverse events, both arrhythmic and HF-related. Indeed, albeit most of cases show a benign course with a life expectancy equal to the general population [1], the SCD remains a rare but devastating events which is still the leading cause of death in young population and athletes [2,3,6]. Furthermore, proper due to the improvement in the HCM management, both pharmacological and non-pharmacological (i.e. ICD, myectomy, LVAD/heart transplantation), there is a growing percentage of HCM patients who develop HF as well as HF-related complications [1,5,28]. The present study, as an ancillary result, confirms the abovementioned concern by showing a significant rate of HF-related events (n. 114 events) with a relatively low number of SCD and SCD-equivalent (n. 14 events) at a mid-term follow-up.

Given the wide HCM clinical spectrum, its genetic nature and the need for targeted prevention strategies, previous studies sought to investigate and to weight the influence of sarcomere variants on the HCM clinical phenotype and outcome [7,8-13,16]. Indeed, HCM represents a sarcomeric disease with LP/P variants in *MYBPC3* and *MYH7* genes (thick filaments) together accounting about 50% of the HCM families whereas LP/P variants in *TNNT2*, *TNNI3* (thin filaments) accounting less than 10% [1,6]. In such a context, an old study by Olivotto and colleagues, conducted in the pre-NGS *scenario* on a cohort of 203 HCM patients with a median follow-up of 4.5 years, suggested that HCM patients with a sarcomere variants had a greater probability of a worse outcome (i.e. cardiac death, non-fatal stroke, end-stage progression) compared to those with no sarcomeric variants [8]. A more recent study by Li and colleagues, on a sample of 558 HCM patients with a median follow-up of 4.5 years, demonstrated that LP/P variants were associated to an early disease-onset, to a high burden of established risk factors and, mainly, to a composite HF end-points (i.e. HF-related hospitalization, heart transplantation, HF-related death, progression to an end-stage phase) [10]. Further support to a possible role of genetic testing in the HCM risk stratification comes from Van Velzen and colleagues that, on a population of 626 HCM patients with a long-term follow-up (> 10 years), confirmed that patients with LP/P variants had a more aggressive phenotype (i.e. young age, high prevalence of SCD risk factors) and found an independent association with all-cause mortality, HF-related and SCD mortality [12]. Eventually, the recent SHARE study, conducted on a large cohort of 2,763 HCM patients followed-up for a median of 2.9 years, confirmed that patients carrying sarcomeric LP/P variants had an earlier disease onset and a greater risk of developing the overall composite outcome (SCD or SCD equivalent, LVAD/cardiac transplantation, progression to an end-stage phase, all-cause mortality, atrial fibrillation and stroke) [13]. The present study confirms that HCM patients carrying LP/P variants have a worse clinical feature in terms of disease onset as well as of historical risk factors. However, we proved just a univariate association between the HCM-mutated status and a composite HF end-point. Albeit it remains difficult to make a close comparison with the abovementioned studies because of different methodological approach (i.e. number of genes screened, variants classification, end-points’ construction), the most likely reason underlying this negative datum could be the optimal clinical and functional characterization of our study cohort. Indeed, we specifically challenged prognostic impact of LP/P variants not only with the historical variables but also, and specifically, with the CPET-derived parameters. Thus, we showed originally that patients with LP/P variants, although younger than the counterpart, exhibited also a more severe functional limitation both in terms of peak VO_2_ and ventilatory efficiency values. Furthermore, due to a concomitant blunted increase of SBP during exercise, HCM patients with LP/P variants showed significantly lower values of CP%. Each of the three abovementioned CPET-derived variable has a specific pathophysiological meaning in the HCM patients’ context [29,30] and a possible capability as outcome predictor. Particularly, the pVO_2_ is a multidimensional parameter dependent on cardiac output (heart rate * stroke volume) and artero-venous O_2_ extraction [24,31] and it has been extensively shown to be a strong predictor of poor outcome in HCM [14-18] as well as in HF patients [32,33]. The CP%, according to its formula [23,24], magnifies the prognostic power of the pVO_2_ through the ABPRE [17,18] which, in turn, depends on the intrinsic myocardial function/geometry as well on abnormal peripheral autonomic reflexes [34,35]. Eventually, the ventilatory efficiency (i.e. VE/VCO_2_ slope) has been shown to correlate with pulmonary capillary wedge pressure and left ventricular diastolic properties in HCM [36]. Although usually preserved in HCM patients, the VE/VCO_2_ slope tends to worsen significantly only in the late systolic dysfunction phase but it is conceivable that it could mirror also an early exercise-induced left ventricular diastolic functional derangement [16,30,36,37].

**5. LIMITATIONS**

The relatively small number of patients enrolled, together with the low number of hard events, represents a certain limitation that allows us to speculate about the usefulness of the genetic analysis results in terms of HCM risk prediction when compared to a full clinical assessment. However, it should be noted that our datum does not argue against the overall importance of genetic testing in the HCM management. Indeed, the identification of unaffected mutated relatives can be possible only after the detection of P/LP variants in affected HCM probands by gene sequencing. [38]. Accordingly, thanks to this early diagnosis, a closer clinical follow-up could be chosen for those asymptomatic mutated subjects. Furthermore, molecular analysis of HCM genes remains one of the pivotal approaches in distinguishing the so called HCM phenocopies where an early diagnosis is crucial to manage it optimally. [2,3]. On the other hand, whenever a P/LP variant is not found in HCM patients, the HCM diagnosis should be carefully re-evaluated. Indeed, this category may include not only HCM patients with a not yet identified sarcomeric gene variant but also non-hereditary forms of hypertrophy. Again, concerning to risk stratification, a comprehensive clinical assessment might be essential. Accordingly, although our HCM patients without LP/P variants were older, more often obstructive, hypertensive and diabetic, their CPET-derived data were better than those obtained in the other group, thus being reasonable that a maximal exercise might unmask the limits of a "sick" myocardium characterized by disarray, fibrosis and small vessel disease.

Another limitation that need to be acknowledged is that, apart from the main four genes (*MYBPC3, MYH7, TNNI3, TNNT2*) that have been tested in all patients, other genes known to be HCM-related (i.e. ACTC1, TPM1, TNNC1, or MYL2 and MYL3) have been tested in most but not all patients. However, it should be remarked that these variants are rarely detected in HCM patients [6,7]. Moreover, given the few number of patients carrying variants in thin filaments genes (< 15%) as well patients with multiple variants (6%) in our study sample, our survival analysis considered the overall impact of the LP/P sarcomeric variants without distinguishing the type of compromised filaments or a possible “gene-dosage effect”. Eventually, we found multiple variants in the same gene in 10 patients and in this specific setting, we cannot determinate whether the genetic alterations were on the same chromosome (heterozygous state) or not (compound heterozygous state).

Finally, besides the genetic profile *per se*, we examined the prognostic effect of several clinical and instrumental variables at a single time point. Accordingly, we cannot exclude that changes in some variables, as for instance an upgrading of treatment during follow-up or upcoming risk factors, altered our survival analysis. However, it is reasonable that seriate clinical and functional evaluations in HCM patients at the highest risk could further magnify our findings rather than rebut them.

**6. CONCLUSIONS**

Our findings support the clinical assessment over the genetic analysis in the HCM risk stratification. Indeed, albeit the presence of LP/P variants has been found to be associated with a more aggressive HCM phenotype in terms of early disease-onset, high burden of historical risk factor and, for the first time, poor functional status, the genetic state was associated only in the univariate analysis to a composite HF end-point. Conversely, within a number of clinical and instrumental variables, the present study reaffirms the pivotal role of the variables derived from a CPET assessment.

**REFERENCES**

1. Maron, B.J.; Maron, M.S. Hypertrophic cardiomyopathy. Lancet. 2013;381(9862):242‐255. doi:10.1016/S0140-6736(12)60397-3
2. Gersh, B.J; Maron, B.J.; Bonow, R.O; Dearani, J.A.; Fifer, M.A.; Link, M.S.; Naidu, S.S.; Nishimura, R.A.; Ommen, S.R.; Rakowski, H.; Seidman, C.E.; Towbin, J.A.; Udelson, J.E.; Yancy, C.W.; American College of Cardiology Foundation/American Heart Association Task Force on Practice Guidelines; American Association for Thoracic Surgery; American Society of Echocardiography; American Society of Nuclear Cardiology; Heart Failure Society of America; Heart Rhythm Society; Society for Cardiovascular Angiography and Interventions; Society of Thoracic Surgeons2011 ACCF/AHA Guideline for the Diagnosis and Treatment of Hypertrophic Cardiomyopathy: Executive Summary: A Report of the American College of Cardiology Foundation/American Heart Association Task Force on Practice Guidelines. Circulation. 2011;124(24):2761‐2796. doi:10.1161/CIR.0b013e318223e230
3. Authors/Task Force members; Elliott, P.M.; Anastasakis, A.; Borger, M.A.; Borggrefe, M.; Cecchi, F.; Charron, P.; Hagege, A.A.; Lafont, A.; Limongelli, G.; Mahrholdt, H.; McKenna, W.J.; Mogensen, J.; Nihoyannopoulos, P.; Nistri, S.; Pieper, P.G.; Pieske, B.; Rapezzi, C.; Rutten, F.H.; Tillmanns, C.; Watkins, H. 2014 ESC Guidelines on diagnosis and management of hypertrophic cardiomyopathy: the Task Force for the Diagnosis and Management of Hypertrophic Cardiomyopathy of the European Society of Cardiology (ESC). Eur Heart J. 2014;35(39):2733‐2779. doi:10.1093/eurheartj/ehu284
4. Maron, B.J.; Ommen, S.R.; Semsarian, C.; Spirito, P.; Olivotto, I.; Maron, M.S. Hypertrophic cardiomyopathy: Present and future, with translation into contemporary cardiovascular medicine. J Am Coll Cardiol. 2014;64(1):83‐99. doi:10.1016/j.jacc.2014.05.003
5. Musumeci, M.B.; Russo, D.; Limite, L.R.; Canepa, M.; Tini, G.; Casenghi, M.; Francia, P.; Adduci, C.; Pagannone, E.; Magrì, D.; Volpe, M.; Autore, C. Long-Term Left Ventricular Remodeling of Patients With Hypertrophic Cardiomyopathy. Am J Cardiol. 2018;122(11):1924‐1931. doi:10.1016/j.amjcard.2018.08.041
6. Marian, A.J.; Braunwald, E. Hypertrophic Cardiomyopathy: Genetics, Pathogenesis, Clinical Manifestations, Diagnosis, and Therapy. Circ Res. 2017;121(7):749‐770. doi:10.1161/CIRCRESAHA.117.311059
7. Rubattu, S.; Bozzao, C.; Pennacchini, E.; Pagannone, E.; Musumeci, M.B.; Piane, M.; Germani, A.; Savio, C.; Francia, P.; Volpe, M.; Autore, C.; Chessa, L. A Next-Generation Sequencing Approach to Identify Gene Mutations in Early- and Late-Onset Hypertrophic Cardiomyopathy Patients of an Italian Cohort. Int J Mol Sci. 2016;17(8):1239. Published 2016 Jul 30. doi:10.3390/ijms17081239
8. Olivotto, I.; Girolami, F.; Ackerman, M.J.; Nistri, S.; Bos, J.M.; Zachara, E.; Ommen, S.R.; Theis, J.L.; Vaubel, R.A.; Re, F.; Armentano, C.; Poggesi, C.; Torricelli, F.; Cecchi, F. Myofilament protein gene mutation screening and outcome of patients with hypertrophic cardiomyopathy. Mayo Clin Proc. 2008;83(6):630‐638. doi:10.4065/83.6.630
9. Selvi Rani, D.; Nallari, P.; Dhandapany, P.S.; Rani, J.; Meraj, K.; Ganesan, M.; Narasimhan, C.; Thangaraj, K. Coexistence of Digenic Mutations in Both Thin (TPM1) and Thick (MYH7) Filaments of Sarcomeric Genes Leads to Severe Hypertrophic Cardiomyopathy in a South Indian FHCM. DNA Cell Biol. 2015;34(5):350‐359. doi:10.1089/dna.2014.2650.
10. Li, Q.; Gruner, C.; Chan, R.H.; Care, M.; Siminovitch, K.; Williams, L.; Woo, A.; Rakowski, H. Genotype-positive status in patients with hypertrophic cardiomyopathy is associated with higher rates of heart failure events. Circ Cardiovasc Genet. 2014;7(4):416‐422. doi:10.1161/CIRCGENETICS.113.000331.
11. Lopes, L.R.; Syrris, P.; Guttmann, O.P.; O'Mahony, C.; Tang, H.C.; Dalageorgou, C.; Jenkins, S.; Hubank, M.; Monserrat, L.; McKenna, W.J.; Plagnol, V.; Elliott PM. Novel genotype-phenotype associations demonstrated by high-throughput sequencing in patients with hypertrophic cardiomyopathy. Heart. 2015 Feb;101(4):294-301. doi: 10.1136/heartjnl-2014-306387
12. van Velzen, H.G.; Vriesendorp, P.A.; Oldenburg, R.A.; van Slegtenhorst, M.A.; van der Velden, J.; Schinkel, A.F.L.; Michels, M. Value of Genetic Testing for the Prediction of Long-Term Outcome in Patients With Hypertrophic Cardiomyopathy. Am J Cardiol. 2016;118(6):881‐887. doi:10.1016/j.amjcard.2016.06.038.
13. Ho, C.Y.; Day, S.M.; Ashley, E.A.; Michel, M.; Pereira, A.C.; Jacoby, D.; Cirino, A.L.; Fox, J.C.; Lakdawala, N.K.; Ware, J.S.; Caleshu, C.A.; Helms, A.S.; Colan, S.D.; Girolami, F.; Cecchi, F.; Seidman, C.E.; Sajeev, G.; Signorovitch, J.; Green, E.M.; Olivotto, I. Genotype and Lifetime Burden of Disease in Hypertrophic Cardiomyopathy: Insights from the Sarcomeric Human Cardiomyopathy Registry (SHaRe). Circulation. 2018;138(14):1387‐1398. doi:10.1161/CIRCULATIONAHA.117.033200
14. Masri, A.; Pierson, L.M.; Smedira, N.G.; Agarwal, S.; Lytle, B.W.; Naji, P.; Thamilarasan, M.; Lever, H.M.; Cho, L.S.; Desai, M.Y. Predictors of longterm outcomes in patients with hypertrophic cardiomyopathy undergoing cardiopulmonary stress testing and echocardiography. Am Heart J. 2015;169(5):684‐692.e1. doi:10.1016/j.ahj.2015.02.006
15. Coats, C.J.; Rantell, K.; Bartnik, A.; Patel, A.; Mist, B.; McKenna, W.J.; Elliott, P.M. Cardiopulmonary exercise testing and prognosis in hypertrophic cardiomyopathy. Circ Heart Fail. 2015;8(6):1022‐1031. doi:10.1161/CIRCHEARTFAILURE.114.002248.
16. Finocchiaro, G.; Haddad, F.; Knowles, J.W.; Caleshu, C.; Pavlovic, A.; Homburger, J.; Shmargad, Y.; Sinagra, G.; Magavern, E.; Wong, M.; Perez, M.; Schnittger, I.; Myers, J.; Froelicher, V.; Ashley, E.A.

Cardiopulmonary responses and prognosis in hypertrophic cardiomyopathy: a potential role for comprehensive noninvasive hemodynamic assessment. JACC Heart Fail. 2015;3(5):408‐418. doi:10.1016/j.jchf.2014.11.011.

1. Magrì, D.; Re, F.; Limongelli, G.; Agostoni, P.; Zachara, E.; Correale, M.; Mastromarino, V.; Santolamazza, C.; Casenghi, M.; Pacileo, G.; Valente, F.; Morosin, M.; Musumeci, M.B.; Pagannone, E.; Maruotti, A.; Uguccioni, M.; Volpe, M.; Autore C. Heart Failure Progression in Hypertrophic Cardiomyopathy - Possible Insights From Cardiopulmonary Exercise Testing. Circ J. 2016;80(10):2204‐2211. doi:10.1253/circj.CJ-16-0432
2. Magrì, D.; Limongelli, G.; Re, F.; Agostoni, P.; Zachara, E.; Correale, M.; Mastromarino, V.; Santolamazza, C.; Casenghi, M.; Pacileo, G.; Valente, F.; Musumeci, B.; Maruotti, A.; Volpe, M.; Autore, C. Cardiopulmonary exercise test and sudden cardiac death risk in hypertrophic cardiomyopathy. Heart. 2016 Apr;102(8):602-9. doi: 10.1136/heartjnl-2015-308453
3. Lang, R.M.; Badano, L.P.; Mor-Avi, V.; Afilalo, J.; Armstrong, A.; Ernande, L.; Flachskampf, F.A.; Foster, E.; Goldstein, S.A.; Kuznetsova, T.; Lancellotti, P.; Muraru, D.; Picard, M.H.; Rietzschel, E.R.; Rudski, L.; Spencer, K.T.; Tsang, W.; Voigt, J.U. Recommendations for cardiac chamber quantification by echocardiography in adults: An update from the American Society of Echocardiography and the European Association of Cardiovascular Imaging. J Am Soc Echocardiogr. 2015 Jan;28(1):1-39.e14. doi: 10.1016/j.echo.2014.10.003
4. Autore, C.; Bernabò, P.; Barillà, C.S.; Bruzzi, P.; Spirito, P. The prognostic importance of left ventricular outflow obstruction in hypertrophic cardiomyopathy varies in relation to the severity of symptoms. J Am Coll Cardiol. 2005;45(7):1076‐1080. doi:10.1016/j.jacc.2004.12.067.
5. Agostoni, P.; Bianchi, M.; Moraschi, A.; Palermo, P.; Cattadori, G.; La Gioia, R.; Bussotti, M.; Wasserman, K. Work-rate affects cardiopulmonary exercise test results in heart failure. Eur J Heart Fail. 2005;7(4):498‐504. doi:10.1016/j.ejheart.2004.06.007
6. Magrì, D.; Agostoni, P.; Sinagra, G.; Re, F.; Correale, M.; Limongelli, G.; Zachara, E.; Mastromarino, V.; Santolamazza, C.; Casenghi, M.; Pacileo, G.; Valente, F.; Morosin, M.; Musumeci, B.; Pagannone, E.; Maruotti, A.; Uguccioni, M.; Volpe, M.; Autore, C. Clinical and prognostic impact of chronotropic incompetence in patients with hypertrophic cardiomyopathy. Int J Cardiol. 2018;271:125‐131. doi:10.1016/j.ijcard.2018.04.019
7. Corrà, U.; Mezzani, A.; Giordano, A.; Bosimini, E.; Giannuzzi, P. Exercise haemodynamic variables rather than ventilatory efficiency indexes contribute to risk assessment in chronic heart failure patients treated with carvedilol. Eur Heart J. 2009;30(24):3000‐3006. doi:10.1093/eurheartj/ehp138
8. Wasserman, K.; Hansen, J.E.; Sue, D.Y.; Stringer, W.; Whipp, B.J. Normal Values. In: Weinberg R, editor. Principles of Exercise Testing and Interpretation. 4th ed Lippincott Williams and Wilkins; Philadelphia: 2005. pp. 160–82.
9. Richards, S.; Aziz, N.; Bale, S.; Bick, D.; Das, S.; Gastier-Foster, J.; Grody, W.W.; Hegde, M.; Lyon, E.; Spector, E.; Voelkerding, K.; Rehm, H.L. and Committee ALQA. Standards and guidelines for the interpretation of sequence variants: a joint consensus recommendation of the American College of Medical Genetics and Genomics and the Association for Molecular Pathology. Genet Med. 2015;17(5):405‐424. doi:10.1038/gim.2015.30.
10. O'Mahony, C.; Jichi, F.; Pavlou, M.; Monserrat, L.; Anastasakis, A.; Rapezzi, C.; Biagini, E.; Gimeno, J.R.; Limongelli, G.; McKenna, W.J.; Omar, R.Z.; Elliott, P.M.; Hypertrophic Cardiomyopathy Outcomes Investigators. A novel clinical risk prediction model for sudden cardiac death in hypertrophic cardiomyopathy (HCM Risk-SCD). Eur Heart J. 2014 Aug 7;35(30):2010-20. doi: 10.1093/eurheartj/eht439.
11. O'Mahony, C.; Akhtar, M. M.; Anastasiou, Z.; Guttmann, O.P.; Vriesendorp, P.A.; Michels, M.; Magrì, D.; Autore, C.; Fernández, A.; Ochoa, J.P.; Leong, K.M.W.; Varnava, A.M.; Monserrat, L.; Anastasakis, A.; Garcia-Pavia, P.; Rapezzi, C.; Biagini, E.; Gimeno, J.R.; Limongelli, G.; Omar, R.Z.; Elliott, P.M. Effectiveness of the 2014 European Society of Cardiology guideline on sudden cardiac death in hypertrophic cardiomyopathy: a systematic review and meta-analysis. Heart. 2019;105(8):623‐631. doi:10.1136/heartjnl-2018-313700
12. Musumeci, M.B.; Mastromarino, V.; Casenghi, M.; Tini, G.; Francia, P.; Maruotti, A.; Romaniello, A.; Magrì, D.; Lillo, R.; Adduci, C.; Volpe, M.; Autore, C. Pulmonary hypertension and clinical correlates in hypertrophic cardiomyopathy. Int J Cardiol. 2017;248:326‐332. doi:10.1016/j.ijcard.2017.07.010
13. Magrì, D.; Santolamazza, C. Cardiopulmonary Exercise Test in Hypertrophic Cardiomyopathy. Ann Am Thorac Soc. 2017;14(Supplement_1):S102‐S109. doi:10.1513/AnnalsATS.201611-884FR
14. Magrì, D.; Agostoni, P.; Cauti, F.M.; Musumeci, B.; Egidy Assenza, G.; De Cecco, C.N.; Muscogiuri, G.; Maruotti, A.; Ricotta, A.; Pagannone, E; Marino, L.; Santini, D.; Proietti, G.; Serdoz, A.; Paneni, F.; Volpe, M.; Autore, C. Determinants of peak oxygen uptake in patients with hypertrophic cardiomyopathy: a single-center study. Intern Emerg Med. 2014;9(3):293‐302. doi:10.1007/s11739-012-0866-x
15. Magrì, D. Peak oxygen uptake in heart failure: Look behind the number! Eur J Prev Cardiol. 2018;25(18):1934‐1936. doi:10.1177/2047487318806987
16. Agostoni, P.; Corrà, U.; Cattadori, G.; Veglia, F.; La Gioia, R.; Scardovi, A.B.; Emdin, M.; Metra, M.; Sinagra, G.; Limongelli, G.; Raimondo, R.; Re, F.; Guazzi, M.; Belardinelli, R.; Parati, G.; Magrì, D.; Fiorentini, C.; Mezzani, A.; Salvioni, E.; Scrutinio, D.; Ricci, R.; Bettari, L.; Di Lenarda, A.; Pastormerlo, L.E.; Pacileo, G.; Vaninetti, R.; Apostolo, A.; Iorio, A.; Paolillo, S.; Palermo, P.; Contini ,M.; Confalonieri, M.; Giannuzzi, P.; Passantino, A.; Cas, L.D.; Piepoli, M.F.; Passino, C.; MECKI Score Research Group Metabolic exercise test data combined with cardiac and kidney indexes, the MECKI score: a multiparametric approach to heart failure prognosis. Int J Cardiol. 2013;167(6):2710‐2718. doi:10.1016/j.ijcard.2012.06.113
17. Paolillo, S.; Veglia, F.; Salvioni, E.; Corrà, U.; Piepoli, M.; Lagioia, R.; Limongelli, G.; Sinagra, G.; Cattadori, G.; Scardovi, A.B.; Metra, M.; Senni, M.; Bonomi, A.; Scrutinio, D.; Raimondo, R.; Emdin, M.; Magrì, D.; Parati, G.; Re, F.; Cicoira, M.; Minà, C.; Correale, M.; Frigerio, M.; Bussotti, M.; Battaia, E.; Guazzi, M.; Badagliacca, R.; Di Lenarda, A.; Maggioni, A.; Passino, C.; Sciomer, S.; Pacileo, G.; Mapelli, M.; Vignati, C.; Clemenza, F.; Binno, S.; Lombardi, C.; Filardi, P.P.; Agostoni, P; MECKI Score Research Group. Heart failure prognosis over time: how the prognostic role of oxygen consumption and ventilatory efficiency during exercise has changed in the last 20 years. Eur J Heart Fail. 2019;21(2):208‐217. doi:10.1002/ejhf.1364
18. Ciampi, Q.; Betocchi, S.; Lombardi, R.; Manganelli, F.; Storto, G.; Losi, M.A.; Pezzella, E.; Finizio, F.; Cuocolo, A.; Chiariello, M. Hemodynamic determinants of exercise-induced abnormal blood pressure response in hypertrophic cardiomyopathy. J Am Coll Cardiol. 2002;40(2):278‐284. doi:10.1016/s0735-1097(02)01950-2
19. Kawasaki, T.; Azuma, A.; Kuribayashi, T.; Akakabe, Y.; Yamano, M.; Miki, S.; Sawada, T.; Kamitani, T.; Matsubara, H.; Sugihara, H. Vagal enhancement due to subendocardial ischemia as a cause of abnormal blood pressure response in hypertrophic cardiomyopathy. Int J Cardiol. 2008;129(1):59‐64. doi:10.1016/j.ijcard.2007.05.023
20. Arena, R.; Owens, D.S.; Arevalo, J.; Smith, K.; Mohiddin, S.A.; McAreavey, D.; Ulisney, K.L.; Tripodi, D.; Fananapazir, L.; Plehn, J.F. Ventilatory efficiency and resting hemodynamics in hypertrophic cardiomyopathy. Med Sci Sports Exerc. 2008 May;40(5):799-805. doi: 10.1249/MSS.0b013e31816459a1
21. Salvioni, E.; Corrà, U.; Piepoli, M.; Rovai, S.; Correale, M.; Paolillo, S.; Pasquali, M.; Magrì, D.; Vitale, G.; Fusini, L.; Mapelli, M.; Vignati, C.; Lagioia, R.; Raimondo, R.; Sinagra, G.; Boggio, F.; Cangiano, L.; Gallo, G.; Magini, A.; Contini, M.; Palermo, P.; Apostolo, A.; Pezzuto, B.; Bonomi, A.; Scardovi, A.B.; Filardi, P.P.; Limongelli, G.; Metra, M.; Scrutinio, D.; Emdin, M.; Piccioli, L.; Lombardi, C.; Cattadori, G.; Parati, G.; Caravita, S.; Re, F.; Cicoira, M.; Frigerio, M.; Clemenza, F.; Bussotti, M.; Battaia, E.; Guazzi, M.; Bandera, F.; Badagliacca, R.; Di Lenarda, A.; Pacileo, G.; Passino, C.; Sciomer, S.; Ambrosio, G.; Agostoni, P; MECKI score research group. Gender and age normalization and ventilation efficiency during exercise in heart failure with reduced ejection fraction. ESC Heart Fail. 2020;7(1):371‐380. doi:10.1002/ehf2.12582
22. Maron, B.J.; Maron, M.S.; Semsarian, C. Genetics of hypertrophic cardiomyopathy after 20 years: clinical perspectives. J Am Coll Cardiol. 2012;60(8):705‐715. doi:10.1016/j.jacc.2012.02.068

**FIGURES’ LEGENDS**

**Figure 1.** Diagram showing the step-by-step screening procedures of the hypertrophic cardiomyopathy (HCM) population considered in our study. LP/P: likely pathogenic/pathogenic sarcomeric variants; VUS: variant of uncertain significance; CV: cardiac events; HF: heart failure; SCD: sudden cardiac death.

**Figure 2.** Top panel: results of genetic testing analysis in the overall study sample. Bottom panel: type and distribution of likely pathogenic/pathogenic (LP/P) variants and variants of uncertain significance (VUS).

**Figure 3.** Kaplan-Meier estimator of survival free from heart failure (HF) (left panel) and sudden cardiac death (SCD) related events according to the presence of likely pathogenic/pathogenic (LP/P) sarcomeric variants.

**TABLE 1.** Main clinical variables of the entire study sample at the study run-in (N: 371 patients)

| **General data** |  |
| --- | --- |
| Age, years | 49±16 |
| Male, n (%) | 238 (64) |
| Age at diagnosis, years | 40±19 |
| NYHA III-IV, n (%) | 24 (6) |
| LVOT obstruction, n (%) | 125 (33) |
| ICD, n (%) | 43 (12) |
| Previous myectomy, n (%) | 20 (5) |
| **SCD risk factors** |  |
| NSVT, n (%) | 121 (32) |
| FH-SCD, n (%) | 48 (13) |
| MWT > 30 mm, n (%) | 26 (7) |
| Unexplained syncope, n (%) | 56 (15) |
| ABPRE, n (%) | 45 (16) |
| **Echocardiographic data** |  |
| LVEDd, mm | 45±5 |
| LAd, mm | 43±7 |
| MWT, mm | 20±5 |
| LVOTG_max_, mm Hg | 11 [6-39] |
| LVEF, % | 62±7 |
| **CPET data** |  |
| Peak HR, % of predicted | 79±13 |
| Peak VO_2_, ml/kg/min | 23±7 |
| Peak VO_2_, % of predicted | 77±18 |
| CP%, % of predicted*mm Hg | 12,937±4,300 |
| VE/VCO_2_ slope | 28.4±5.5 |
| **Medical treatment** |  |
| β-blocker, n (%) | 228 (61) |
| Non dihydropyridine CCB, n (%) | 31 (8) |
| ACE-I/ARB, n (%) | 107 (29) |
| Diuretics, n (%) | 78 (21) |
| Cordarone, n (%) | 33 (9) |

Data are expressed as mean ± SD, as absolute number of patients (% on total sample) or as median [25^th^-75^th^ percentile]. VUS.: variant of uncertain significance; LP: likely pathogenic; P: pathogenic; NYHA: New York Heart Association; ICD: implantable cardioverter defibrillator; SCD: sudden cardiac death; NSVT: non-sustained ventricular tachycardia; FH: family history; ABPRE: abnormal blood pressure response at exercise; LVEDd: left ventricular end diastolic diameter; LAd: left atrial diameter; MWT: maximum wall thickness; LVOTG_max_: maximal LV outflow tract gradient; LVEF: LV ejection fraction; ΔSBP: difference between peak and resting systolic blood pressure; HR: heart rate; VO_2_: oxygen uptake; CP: circulatory power; VE/VCO_2_ slope: relation between ventilation versus carbon dioxide production; CCB: calcium channel blocker; ACE-I/ARB: angiotensin converting enzyme inhibitors / angiotensin receptor blocker

**TABLE 2.** Main clinical variables of the study sample at the study run-in according to genetic testing results.

| **General data** | **No Variants and VUS**  **(n:168)** | **LP/P Variants**  **(n:203)** | | **P values** |
| --- | --- | --- | --- | --- |
| Age, years | 53±18 | 45±16 | <0.001 | |
| Male, n (%) | 112 (67) | 124(61) | NS | |
| Age at diagnosis, years | 47±20 | 35±17 | <0.001 | |
| NYHA III-IV, n (%) | 10 (6) | 14 (7) | NS | |
| LVOT obstruction, n (%) | 79 (47) | 45 (22) | <0.001 | |
| ICD, n (%) | 11 (6) | 32 (16) | 0.019 | |
| Previous myectomy, n (%) | 9 (5) | 11 (5) | NS | |
| **SCD risk factors** |  |  |  | |
| NSVT, n (%) | 48 (28) | 73 (36) | NS | |
| FH-SCD, n (%) | 16 (9) | 32 (16) | 0.049 | |
| MWT > 30 mm, n (%) | 10 (6) | 16 (8) | NS | |
| Unexplained syncope, n (%) | 29 (17) | 26 (13) | NS | |
| ABPRE, n (%) | 11 (7) | 34 (17) | 0.003 | |
| **Echocardiographic data** |  |  |  | |
| LVEDd, mm | 46±4 | 45±6 | NS | |
| LAd, mm | 43±7 | 43±7 | NS | |
| MWT, mm | 20±5 | 20±5 | NS | |
| LVOTG_max_, mm Hg | 16 [9-39] | 10 [5-33] | 0.023 | |
| LVEF, % | 63±4 | 61±6 | <0.001 | |
| **CPET data** |  |  |  | |
| Peak HR, % of predicted | 78±12 | 79±13 | NS | |
| Peak VO_2_, ml/kg/min | 23±7 | 23±7 | NS | |
| Peak VO_2_, % of predicted | 79±18 | 75±18 | 0.032 | |
| CP%, % of predicted*mm Hg | 14,070±4,269 | 12,015±4,020 | <0.001 | |
| VE/VCO_2_ slope | 27.5±4.9 | 29.1±6.0 | 0.019 | |
| **Medical treatment** |  |  |  | |
| β-blocker, n (%) | 105 (62) | 122 (60) | NS | |
| Non dihydropyridine CCB, n (%) | 11 (7) | 20 (10) | NS | |
| ACE-I/ARB, n (%) | 58 (34) | 48 (24) | 0.014 | |
| Diuretics, n (%) | 37 (22) | 41 (21) | NS | |
| Cordarone, n (%) | 16 (9) | 17 (8) | NS | |

Data are expressed as mean ± SD, as absolute number of patients (% on total sample) or as median [25^th^-75^th^ percentile]. See table 1 for other abbreviations.

**TABLE 3.** Main significant univariate Cox proportional survival analysis according to the main variables for the two main study end-points.

|  | **HF endpoint (n: 52)** | | | | | **SCD endpoint (n: 14)** | | | | | | | | |
| --- | --- | --- | --- | --- | --- | --- | --- | --- | --- | --- | --- | --- | --- | --- |
|  | | **H.R. (95% C.I.)** | **P values** | **C-index** | | **H.R. (95% C.I.)** | | | **P values** | | | | **C-index** | |
| **Age at CPET** | | -- | NS | | -- | 0.964 (0.934-0.996) | | | 0.038 | | | 0.613 | | |
| **Male sex** | | -- | NS | | -- | -- | | | NS | | | -- | | |
| **Age at diagnosis** | | -- | NS | | -- | 0.944 (0.906-0.983) | | 0.006 | | | | 0.729 | | |
| **LVOTO** | | 2.110 (1.215-3.664) | <0.01 | | 0.641 | -- | | | NS | | | -- | | |
| **Family history of SD** | | 1.869 (1.010-3.460) | 0.046 | | 0.522 | 2.830 (0.892-8.979) | | | 0.077 | | | 0.607 | | |
| **Unexplained Syncope** | | -- | NS | | -- | -- | | | NS | | | -- | | |
| **NSVT** | | 1.917 (1.102-3.333) | 0.021 | | 0.548 | -- | | | NS | -- | | | | |
| **ABPRE** | | 3.418 (1.769-6.605) | <0.001 | | 0.641 | -- | | | NS | | | -- | | |
| **MWT > 30 mm** | | -- | NS | | -- | 3.956 (1.210-12.940) | | | 0.023 | | 0.569 | | | |
| **MWT** | | -- | NS | | -- | 1.100 (1.012-1.195) | | | 0.025 | | 0.593 | | | |
| **LAd** | | 1.077 (1.039-1.116) | <0.001 | | 0.704 | 1.054 (0.984-1.129) | | | 0.112 | | 0.660 | | | |
| **LVOTG_max_** | | 1.016 (1.008-1.024) | <0.001 | | 0.672 | 0.971 (0.937-1.007) | | | 0.123 | | 0.577 | | | |
| **LVEF** | | 0.929 (0.899-0.959) | <0.001 | | 0.587 | -- | | NS | | -- | | | | |
| **pVO_2_, ml/Kg/min** | | 0.851 (0.799-0.905) | <0.001 | | 0.739 | -- | | | NS | | -- | | | |
| **pVO_2_, % of predicted** | | 0.851 (0.799-0.905) | <0.001 | | 0.749 | -- | | | NS | | -- | | | |
| **VE/VCO_2_ slope** | | 1.017 (1.069-1.146) | <0.001 | | 0.724 | -- | NS | | | | | | | -- |
| **CP%** | | 0.998 (0.997-0.999) | <0.001 | | 0.778 | 0.998 (0.997-1.000) | 0.052 | | | | | | | 0.705 |
| ***LP or P variants*** | | 2.395 (1.171-4.856) | 0.013 | | 0.609 | -- | NS | | | | | | | -- |

H.R.: hazard ratio; C.I.: confidence interval. See table 1 for other abbreviations.

**TABLE 4.** Significant multivariate Cox proportional survival analysis and test for proportional hazards assumption for the two study end-points.

|  | **Multivariate Cox Proportional Survival Analysis**   \| **HF endpoint** \| **SCD endpoint** \| \| --- \| --- \| | | | | | |
| --- | --- | --- | --- | --- | --- | --- | --- | --- |
|  | **H.R. (95% C.I.)** | **P values** | **C-index** | **H.R. (95% C.I.)** | **P values** | **C-index** |
| **LAd** | 1.083 (1.039- 1.130) | <0.001 | 0.839 | 1.078 (1.005-1.163) | 0.0485 | 0.738 |
| **CP%** | 0.998 (0.997- 0.999) | <0.001 |  | 0.998 (0.9996- 1.000) | 0.0488 |  |
| **VE/VCO_2_ slope** | 1.044 (0.999-1.090) | 0.05 |  |  |  |  |

See table 1 and 2 for other abbreviations.
