# Supplementary material for: Risk Stratification in Hypertrophic Cardiomyopathy. Insights from Genetic Analysis and Cardiopulmonary Exercise Testing
Source: J Clin Med. 2020 May 28;9(6):1636. doi: 10.3390/jcm9061636 (PMC7356142; doi:10.3390/jcm9061636)
Supplement: Supplementary file 1 [file jcm-09-01636-s001.zip › Genetics Vs CPET in HCM/Tables_Supplementary.docx]

**Supplemental Table 1.** List of pathogenic (P) and likely pathogenic (LP) gene variants identified

| ***gene*** | ***locus*** | ***dbSNP*** | ***cDNA***  ***(HGVS)*** | ***protein*** | ***mutation*** | ***GnomAD*** | ***ACMG***  ***class*** | ***N*** |
| --- | --- | --- | --- | --- | --- | --- | --- | --- |
| ***MYBPC3*** | chr11:47372807 | / | c.275_276del | p.(Leu92GInfs*20) | frameshift | / | *P* | 1 |
| ***MYBPC3*** | chr11:47371628 | rs397516050 | c.442G>A | p.(Gly148Arg) | missense | 0.0000637 | *LP* | 3 |
| ***MYBPC3*** | chr11:47371426 | rs375607980 | c.553A>T | p.(Lys185*) | nonsense | / | *P* | 1 |
| ***MYBPC3*** | chr11:47370085 | / | c.659_662del | p.(Tyr220Cysfs*79) | frameshift | / | *P* | 1 |
| ***MYBPC3*** | chr11:47369975 | rs397516074 | c.772G>A | p.(Glu258Lys) | missense | 0.0000166 | *LP* | 6 |
| ***MYBPC3*** | chr11:47369442 | rs373730381 | c.787G>A | p.(Gly263Arg) | missense | 0.0000926 | *LP* | 1 |
| ***MYBPC3*** | chr11:47369407 | rs397516073 | c.821+1G>A | p? | splicing | 0.0000289 | *P* | 1 |
| ***MYBPC3*** | chr11:47368189 | rs397516080 | c.913_914del | p.(Phe305Profs*27) | frameshift | / | *P* | 2 |
| ***MYBPC3*** | chr11:47367923 | rs397516082 | c.927-2A>G | p? | splicing | 0.00000508 | *P* | 1 |
| ***MYBPC3*** | chr11:47367758 | rs794727046 | c.1090G>A | p.(Ala364Thr) | missense | / | *LP* | 1 |
| ***MYBPC3*** | chr11:47367757 | rs727504269 | c.1090+1G>A | p.? | splicing | / | *P* | 1 |
| **MYBPC3** | chr11:47365154 | rs397515887 | c.1112C>G | p.(Pro371Arg) | missense | / | *LP* | 4 |
| ***MYBPC3*** | chr11:47365147 | / | c.1119C>G | p.(Tyr373*) | nonsense | / | *P* | 1 |
| ***MYBPC3*** | chr11:47364465 | rs374255707 | c.1373G>A | p.(Arg458His) | missense | 0.000049 | *LP* | 1 |
| ***MYBPC3*** | chr11:47364429 | rs776734314 | c.1409G>A | p.(Arg470Gln) | missense | 0.00000405 | *LP* | 1 |
| ***MYBPC3*** | chr11:47364296 | rs397515903 | c.1458-1G>A | p? | splicing | / | *P* | 1 |
| ***MYBPC3*** | chr11:47364297 | / | c.1458-2A>G | p.? | splicing | / | *P* | 1 |
| ***MYBPC3*** | chr11:47364282 | rs730880543 | c.1471G>A | p.(Val491Met) | missense | 0.0000562 | *LP* | 1 |
| ***MYBPC3*** | chr11:47364270 | rs397515905 | c.1483C>T | p.(Arg495Trp) | missense | / | *P* | 1 |
| ***MYBPC3*** | chr11:47364248 | rs397515907 | c.1505G>A | p.(Arg502Gln) | missense | / | *P* | 10 |
| ***MYBPC3*** | chr11:47364162 | rs397515912 | c.1591G>C | p.(Gly531Arg) | missense | 0.0000121 | *LP* | 6 |
| ***MYBPC3*** | chr11:47364129 | rs121909374 | c.1624G>C | p.(Glu542Gln) | missense | 0.000013 | *P* | 2 |
| ***MYBPC3*** | chr11:47364125 | rs397515916 | c.1624+4A>T | p.? | intronic | 0.0000133 | *LP* | 1 |
| ***MYBPC3*** | chr11:47362773 | / | c.1813G>C | p.(Lys605His) | missense | / | *LP* | 3 |
| ***MYBPC3*** | chr11:47361267 | rs730880561 | c.2002C>T | p.(Arg668Cys) | missense | 0.000012 | *LP* | 1 |
| ***MYBPC3*** | chr11:47361239 | rs786204345 | c.2030C>T | p.(Pro677Leu) | missense | / | *LP* | 1 |
| ***MYBPC3*** | chr11:47360197 | rs397515954 | c.2182G>T | p.(Glu728*) | nonsense | / | *P* | 1 |
| ***MYBPC3*** | chr11:47360181 | rs534345197 | c.2198G>A | p.(Arg733His) | missense | 0.0000523 | *LP* | 1 |
| ***MYBPC3*** | chr11:47360120 | rs774521272 | c.2258dup | p.(Lys754Glu*79) | frameshift | 0.00000803 | *P* | 2 |
| ***MYBPC3*** | chr11:47360071 | rs36211723 | c.2308G>A | p.(Asp770Asn) | missense | 0.0000161 | *P* | 5 |
| ***MYBPC3*** | chr11:47359347 | rs11729952 | c.2309-2A>G | p.? | splicing | / | *P* | 14 |
| ***MYBPC3*** | chr11:47359343 | rs371488302 | c.2311G>A | p.(Val771Met) | missense | 0.0000396 | *LP* | 1 |
| ***MYBPC3*** | chr11:47359115 | rs375675796 | c.2429G>A | p.(Arg810His) | missense | 0.000048 | *LP* | 6 |
| ***MYBPC3*** | chr11:47359109 | rs786204350 | c.2435A>G | p.(Tyr818*) | nonsense | / | *LP* | 1 |
| ***MYBPC3*** | chr11:47359085 | rs2856655 | c.2459G>A | p.(Arg820Gln) | missense | 0.0000201 | *P* | 2 |
| ***MYBPC3*** | chr11:47357511 | rs397515981 | c.2654 C>T | p.(Thr885Met) | missense | 0.0000152 | *LP* | 1 |
| ***MYBPC3*** | chr11:47357476 | / | c.2689_2690insCCTGGCTCTGGCTACAGCA | p.(Gly897Alafs*160) | frameshift | / | *P* | 1 |
| ***MYBPC3*** | chr11:47357472 | / | c.2692_2693del | p.(Ala898Argfs*152) | frameshift | / | *P* | 2 |
| ***MYBPC3*** | chr11:47357448 | / | c.2717T>G | p.(Val906Gly) | missense | / | *LP* | 1 |
| ***MYBPC3*** | chr11:47356671 | rs387907267 | c.2827C>T | p.(Arg943*) | nonsense | 0.0000121 | *P* | 1 |
| ***MYBPC3*** | chr11:473556652 | rs786204352 | c.2846dup | p.(Met949llefs*102) | frameshift | / | *P* | 2 |
| ***MYBPC3*** | chr11:47356593 | rs397515992 | c.2905C>T | p.(Gln969*) | nonsense | / | *P* | 1 |
| ***MYBPC3*** | chr11:47356592 | rs397515991 | c.2905+1G>A | p.? | splicing | / | *P* | 1 |
| ***MYBPC3*** | chr11:47355472 | / | c.2994 +1G>A | p.? | splicing | / | *P* | 1 |
| ***MYBPC3*** | chr11:47355264 | rs730880586 | c.3034C>T | p.(Gln1012*) | nonsense | / | *P* | 4 |
| ***MYBPC3*** | chr11:47354882 | rs397516007 | c.3192dup | p.(Lys1065Glnfs*12) | frameshift | / | *P* | 10 |
| ***MYBPC3*** | chr11:47354824 | / | c.3251T>C | p.(Leu1084Pro) | missense | / | *LP* | 2 |
| ***MYBPC3*** | chr11:47354791 | rs755653624 | c.3284C>T | p.(Thr1095Met) | missense | 0.0000242 | *LP* | 1 |
| ***MYBPC3*** | chr11:47354209 | rs199669878 | c.3357C>A | p.(Tyr1119*) | nonsense | / | *P* | 1 |
| ***MYBPC3*** | chr11:47354491 | rs996588721 | c.3364A>T | p.(Ther112Ser) | missense | / | *LP* | 1 |
| ***MYBPC3*** | chr11:47354404 | / | c.3450_3451del | p.(Arg1150Serfs*18) | frameshift | / | *P* | 2 |
| ***MYBPC3*** | chr11:47354130 | rs730880596 | c.3614G>C | p.(Arg1205Pro) | missense | / | *LP* | 1 |
| ***MYBPC3*** | chr11:47354126 | rs1060501484 | c.3617del | p.(Gly1206Valfs*31) | frameshift | / | *P* | 1 |
| ***MYBPC3*** | chr11:47354127 | rs1057517769 | c.3617G>A | p.(Gly1206Asp) | missense | / | *LP* | 1 |
| ***MYBPC3*** | chr11:47353801 | / | c.3636T>G | p.(Ile1212Met) | missense | / | *LP* | 1 |
| ***MYBPC3*** | chr11:47353740 | rs397516037 | c.3697C>T | p.(Gln1233*) | nonsense | 0.00000802 | *P* | 1 |
| ***MYBPC3*** | chr11:47353662 | rs730880605 | c.3775C>T | p.(Gln1259*) | nonsense | / | *P* | 2 |
| ***MYH7*** | chr14:23901922 | rs397516209 | c.428G>A | p.(Arg143Gln) | missense | 0.00000398 | *P* | 4 |
| ***MYH7*** | chr14:23900850 | rs1057517773 | c.676G>A | p.(Ala226Thr) | missense | / | *LP* | 2 |
| ***MYH7*** | chr14:23900677 | rs3218713 | c.746G>A | p.(Arg249Gln) | missense | / | *P* | 1 |
| ***MYH7*** | chr14:23899843 | rs730880923 | c.925G>A | p.(Asp309Asn) | missense | 0.0000159 | *LP* | 1 |
| ***MYH7*** | chr14:23898464 | rs730880868 | c.1231G>A | p.(Val411Ile) | missense | 0.0000159 | *LP* | 2 |
| ***MYH7*** | chr14:23898195 | / | c.1376T>C | p.(Val459Ala) | missense | / | *LP* | 1 |
| ***MYH7*** | chr14:23897738 | rs727504237 | c.1549C>A | p.(Leu517Met) | missense | / | *LP* | 5 |
| ***MYH7*** | chr14:23897067 | rs730880930 | c.1615A>C | p.(Met539Leu) | missense | / | *LP* | 4 |
| ***MYH7*** | chr14:23896866 | c.1816G>A | c.1816G>A | p.(Val606Met) | missense | 0.00000398 | *P* | 5 |
| ***MYH7*** | chr14:23896043 | rs397516127 | c.1987C>A | p.(Arg663Ser) | missense | / | *P* | 1 |
| ***MYH7*** | chr14:23896042 | rs371898076 | c.1988G>A | p.(Arg663His) | missense | 0.00000795 | *P* | 3 |
| ***MYH7*** | chr14:23895254 | rs886039030 | c.2081G>A | p.(Arg694His) | missense | 0.00000398 | *LP* | 2 |
| ***MYH7*** | chr14:23895189 | rs121913638 | c.2146G>A | p.(Gly716Arg) | missense | / | *P* | 3 |
| ***MYH7*** | chr14:23895180 | rs121913637 | c.2155C>T | p.(Arg719Trp) | missense | / | *P* | 2 |
| ***MYH7*** | chr14:23895179 | rs121913641 | c.2156G>A | p.(Arg719Gln) | missense | / | *P* | 2 |
| ***MYH7*** | chr14:23895023 | rs121913630 | c.2167C>T | p.(Arg723Cys) | missense | 0.0000119 | *P* | 5 |
| ***MYH7*** | chr14:23894983 | rs727503261 | c.2207T>C | p.(Ile736Thr) | missense | / | *P* | 2 |
| ***MYH7*** | chr14:23894969 | rs121913632 | c.2221G>C | p.(Gly741Arg) | missense | / | *P* | 1 |
| ***MYH7*** | chr14:23894568 | rs730880736 | c.2346C>A | p.(Ser782Arg) | missense | / | *LP* | 1 |
| ***MYH7*** | chr14:23894525 | rs3218716 | c.2389G>A | p.(Ala797Thr) | missense | 0.0000239 | *LP* | 2 |
| ***MYH7*** | chr14:23894121 | / | c.2536G>A | p.(Glu846Lys) | missense | / | *LP* | 3 |
| ***MYH7*** | chr14:23894116 | rs397516155 | c.2539_2541del | p.(Lys847del) | inframe | / | *P* | 2 |
| ***MYH7*** | chr14:23894051 | rs202141173 | c.2606G>A | p.(Arg869His) | missense | 0.0000239 | *LP* | 4 |
| ***MYH7*** | chr14:23894007 | / | c.2650A>T | p.(Lys884*) | nonsense | / | *P* | 1 |
| ***MYH7*** | chr14:23893259 | rs397516170 | c.2779G>A | p.(Glu927Lys) | missense | / | *LP* | 1 |
| ***MYH7*** | chr14:23893250 | rs397516171 | c.2788G>C | p.(Glu930Gln) | missense | / | *P* | 2 |
| ***MYH7*** | chr14:23893243 | / | c.2795T>A | p.(Met932Lys) | missense | / | *LP* | 1 |
| ***MYH7*** | chr14:23893234 | rs730880761 | c.2804A>T | p.(Glu935Val) | missense | 0.00000795 | *P* | 1 |
| ***MYH7*** | chr14:23893210 | / | c.2828T>C | p.(Leu943Pro) | missense | / | *LP* | 1 |
| ***MYH7*** | chr14:23891501 | rs45611033 | c.3133C>T | p.(Arg1045Cys) | missense | 0.0000239 | *P* | 3 |
| ***MYH7*** | chr14:23887522 | rs727503246 | c.4066G>A | p.(Glu1356Lys) | missense | / | *P* | 1 |
| ***TNNI3*** | chr19:55665516 | rs121917760 | c.431T>A | p.(Leu144Gln) | missense | / | *LP* | 6 |
| ***TNNI3*** | chr19:55665398 | / | c.549G>T | p.(Lys183Asn) | missense | / | *LP* | 6 |
| ***TNNI3*** | chr19:55663278 | rs397516357 | c.557G>A | p.(Arg186Gln) | missense | / | *P* | 3 |
| ***TNNI3*** | chr19:55663243 | rs727504285 | c.592C>G | p.(Leu198Val) | missense | 0.00000401 | *LP* | 4 |
| ***TNNT2*** | chr1:201334751 | rs397516452 | c.251G>C | p.(Arg84Thr) | missense | / | *LP* | 2 |
| ***TNNT2*** | chr1:201333497 | rs397516463 | c.418C>T | p.(Arg140Cys) | missense | / | *P* | 1 |

*Abbreviations: dbSNP, Single Nucleotide Polymorphism Database (*[*https://www.ncbi.nlm.nih.gov/snp/*](https://www.ncbi.nlm.nih.gov/snp/)*); rs, reference SNP; HGVS: Human Genome Variation Society (*[*http://www.HGVS.org/varnomen)*](http://www.HGVS.org/varnomen))*; GnomAD, Genome Aggregation Database (*[*https://gnomad.broadinstitute.org/*](https://gnomad.broadinstitute.org/)*); ACMG, American College of Medical Genetics and Genomics; P, pathogenic; LP, likely pathogenic. Variants were annotated according to the current HGVS nomenclature using the RefSeq: NM_000256.3 (MYBPC3), NM_000257.4 (MYH7), NM_000363.5 (TNNI3), NM_001276347.6 (TNNT2).*

**Supplemental Table 2.** List of variants of uncertain significance (VUS) identified

| ***gene*** | ***locus*** | ***dbSNP*** | ***c.DNA (HGVS)*** | ***protein*** | ***mutation*** | ***GnomAD*** | ***ACMG***  ***class*** | ***N*** |
| --- | --- | --- | --- | --- | --- | --- | --- | --- |
| ***ACTC1*** | chr15:35083451 | rs869025354 | c.854T>C | p.(Met285Thr) | missense | ***/*** | *VUS* | 3 |
| ***ACTC1*** | chr15:35085615 | / | c.285G>T | p.(Glu95Asp) | missense | ***/*** | *VUS* | 1 |
| ***MYBPC3*** | chr11:47373015 | rs758044508 | c.67G>A | p.(Ala23Thr) | missense | 0.0000295 | *VUS* | 1 |
| ***MYBPC3*** | chr11:47372891 | rs1250637243 | c.191A>G | p.(His64Arg) | missense | 0.00000446 | *VUS* | 2 |
| ***MYBPC3*** | chr11:47372888 | rs753300898 | c.194C>T | p.(Thr65Met) | missense | / | *VUS* | 1 |
| ***MYBPC3*** | chr11:47371343 | rs397516062 | c.636C>G | p.(Ser212Arg) | missense | / | *VUS* | 1 |
| ***MYBPC3*** | chr11:47364602 | rs193922377 | c.1321G>A | p.(Glu441Lys) | missense | 0.000138 | *VUS* | 1 |
| ***MYBPC3*** | chr11:47364129 | rs121909374 | c.1664T>C | p.(Met555Thr) | missense | 0.0000201 | *VUS* | 2 |
| ***MYBPC3*** | chr11:47356628 | rs193922380 | c.2870C>G | p.(Thr957Ser) | missense | 0.00101 | *VUS* | 1 |
| ***MYBPC3*** | chr11:47354209 | rs199669878 | c.3535G>A | p.(Glu1179Lys) | missense | 0.000452 | *VUS* | 1 |
| ***MYH7*** | chr14:23902781 | rs397516117 | c.161G>A | p.(Arg54Gln) | missense | 0.0000239 | *VUS* | 1 |
| ***MYH7*** | chr14:23890202 | rs367546859 | c.3301G>A | p.(Gly1101Ser) | missense | 0.0000597 | *VUS* | 1 |
| ***MYH7*** | chr14:23889413 | / | c.3367G>C | p.(Glu1123Gln) | missense | / | *VUS* | 1 |
| ***MYH7*** | chr14:23887615 | rs1364747700 | c.3973G>A | p.(Ala1325Thr) | missense | 0.000000 | *VUS* | 1 |
| ***MYH7*** | chr14:23886806 | rs397516207 | c.4259G>A | p.(Arg1420Gln) | missense | / | *VUS* | 2 |
| ***MYH7*** | chr14:23886479 | rs876657884 | c.4402G>A | p.(Glu1468Lys) | missense | / | *VUS* | 1 |
| ***MYH7*** | chr14:23885041 | rs397516233 | c.4954G>T | p.(Asp1652Tyr) | missense | 0.0000477 | *VUS* | 1 |
| ***MYH7*** | chr14:23885010 | rs370328209 | c.4985G>A | p.(Arg1662His) | missense | 0.0000597 | *VUS* | 1 |
| ***MYL3*** | chr3: 46899901 | rs145520567 | c.532G>A | p.(Asp178Asn) | missense | 0.0002189 | *VUS* | 1 |
| ***MYL3*** | chr3:46902303 | rs139794067 | c.170C>A | p.(Ala57Asp) | missense | 0.0001710 | *VUS* | 1 |
| ***TPM1*** | chr15:63335110 | rs397516391 | c.82G>C | p.(Asp28His) | missense | 0.0000319 | *VUS* | 1 |
| ***TNNI3*** | chr19:55668965 | rs773513015 | c.-8G>A | p.? | 5'UTR | 0.0000281 | *VUS* | 1 |
| ***TNNI3*** | chr19:55665561 | / | c.386C>G | p.(Thr129Ser) | missense | / | *VUS* | 1 |
| ***TNNI3*** | chr19:55665519 | rs397516348 | c.428C>A | p.Thr143Asn | missense | 0.0000362 | *VUS* | 2 |
| ***TNNT2*** | chr1:201334400 | / | c.330T>G | p.(Phe120Leu) | missense | / | *VUS* | 1 |
| ***TNNT2*** | chr1:201332415 | rs760490476 | c.600+9G>C | p.? | intronic | 0.00000398 | *VUS* | 1 |
| ***TNNT2*** | chr1:201328787 | / | c.785A>T | p.(Asn262Ile) | missense | / | *VUS* | 1 |
| ***TNNT2*** | chr1:201330423 | / | c.794A>T | p.(Lys265Ile) | missense | / | *VUS* | 2 |
| ***TNNT2*** | chr1:201328373 | rs121964857 | c.853C>T | p.(Arg285Cys) | missense | / | *VUS* | 1 |
| ***TNNC1*** | chr3:52485431 | rs730881061 | c.430A>G | p.(Asn144Asp) | missense | / | *VUS* | 1 |

*Abbreviations: dbSNP, Single Nucleotide Polymorphism Database (*[*https://www.ncbi.nlm.nih.gov/snp/*](https://www.ncbi.nlm.nih.gov/snp/)*); rs, reference SNP; HGVS: Human Genome Variation Society (*[*http://www.HGVS.org/varnomen)*](http://www.HGVS.org/varnomen))*; GnomAD, Genome Aggregation Database (*[*https://gnomad.broadinstitute.org/*](https://gnomad.broadinstitute.org/)*); ACMG, American College of Medical Genetics and Genomics; VUS, variant of uncertain significance. Variants were annotated according to the current HGVS nomenclature using the RefSeq: NM_005159.5 (ACTC1), NM_000256.3 (MYBPC3), NM_000257.4 (MYH7), NM_000258.3 (MYL3),* *NM_001018020.2 (TPM1) NM_000363.5 (TNNI3), NM_001276347.6 (TNNT2), NM_003280.3 (TNNC1).*

**Supplemental Table 3.** HCM patients with multiple sarcomeric genes variants (at least one LP/P variants)

| ***GENOTYPE*** | | | | | | | |
| --- | --- | --- | --- | --- | --- | --- | --- |
| ***ID Family*** | ***gender*** | ***MYH7*** | ***MYBPC3*** | ***TNNI3*** | ***TNNT2*** | ***ACTC1*** | ***Clinical Significance*** |
| 30 | M |  | c.442G>A(;)772G>A |  |  |  | *LP/LP* |
| 35 | F | c.1549C>A | c.3364A>T |  |  |  | *LP/LP* |
| 47 | M |  | c.3251T>C | c.428C>A |  |  | *LP/VUS* |
| 47 | M |  | c.3251T>C | c.428C>A |  |  | *LP/VUS* |
| 57 | F |  |  | c.592C>G | c.600+9G>C |  | *LP/VUS* |
| 58 | F | c.2606G>A |  |  |  | c.854T>C | *LP/VUS* |
| 58 | F | c.2606G>A |  |  |  | c.854T>C | *LP/VUS* |
| 58 | M | c.2606G>A |  |  |  | c.854T>C | *LP/VUS* |
| 62 | F | c.2207T>C | c.191A>G |  |  |  | *P/VUS* |
| 62 | M | c.2207T>C | c.191A>G |  |  |  | *P/VUS* |
| 92 | M | c.2788G>C | c.67G>A |  |  |  | *P/VUS* |
| 108 | M |  | c.1112C>G (;)c.3192dup |  |  |  | *LP/P* |
| 117 | M | c.2167C>T | c.3284C>T |  |  |  | *P/LP* |
| 143 | M | c.2804A>T(;)3973G>A |  |  |  |  | *P/VUS* |
| 158 | M |  | c.1591 G> C(;)3636T>G(;)2309-2A>G |  |  |  | *LP/LP/P* |
| 185 | F | c.2167C>T(;)2650A>T |  |  |  |  | *P/P* |
| 195 | M |  | c.1591G>C (;)1112C>G (;)c.3192dup |  |  |  | *LP/LP/P* |
| 207 | F |  | c.1624G>C | c.386C>G |  |  | *P/VUS* |
| 230 | F |  | c.1112C>G (;)c.3192dup |  |  |  | *LP/P* |
| 251 | F | c.1231G>A(;)2606G>A |  |  |  |  | *LP/LP* |
| 262 | M | c.4985G>A | c.913_914del |  |  |  | *VUS/P* |
| 276 | M |  | c.1112C>G (;)c.3192dup |  |  |  | *LP/P* |
| 280 | M |  | c.1321G>A(;)2689_2690insCCTGGCTCTGGCTACAGCA |  |  |  | *VUS/P* |
| 304 | M |  | c.787G>A |  | c.330T>G |  | *LP/VUS* |

*HCM patients with multiple sarcomeric genes variants including double heterozygous for: MYBPC3-MYH7 (n=6), MYH7-TNNI3 (n=3), TNNI3-TNNT2 (n=1), MYBPC3-TNNT2 (n=1), MYH7-ACTC1 (n=3). For the multiple variants identified in MYBPC3 (n=7) and in MYH7 (n=3) the phase could not been identified for the unavailability of relatives. Abbreviations: F: female; M: male; ACTC1: cardiac muscle alpha actin; MYBPC3: myosin-binding protein C; MYH7: beta-myosin heavy chain; TNNI3: cardiac troponin; TNNT2:* *cardiac troponin T; P, pathogenic; LP, likely pathogenic; VUS, variant of uncertain significance.*
