# Supplementary figures and images for: Risk Stratification in Hypertrophic Cardiomyopathy. Insights from Genetic Analysis and Cardiopulmonary Exercise Testing
Source: J Clin Med. 2020 May 28;9(6):1636. doi: 10.3390/jcm9061636 (PMC7356142; doi:10.3390/jcm9061636)

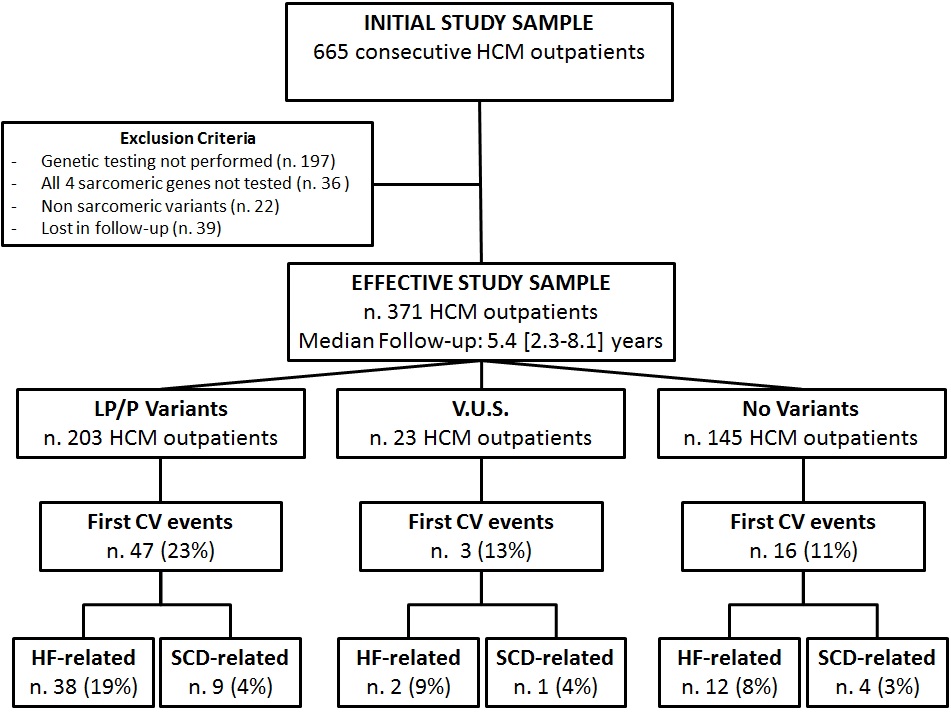

Supplement: Supplementary file 1 [file jcm-09-01636-s001.zip › Genetics Vs CPET in HCM/Figure 1.jpg]

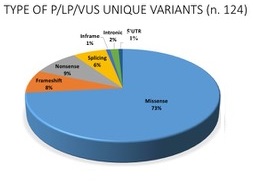

Supplement: Supplementary file 1 [file jcm-09-01636-s001.zip › Genetics Vs CPET in HCM/Figure 1_supplementary.jpeg]

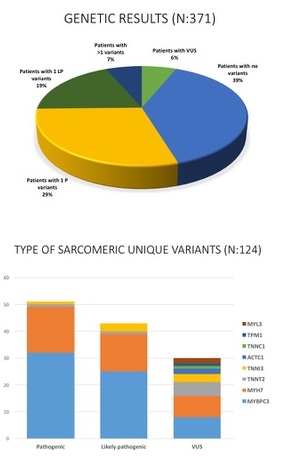

Supplement: Supplementary file 1 [file jcm-09-01636-s001.zip › Genetics Vs CPET in HCM/Figure 2.jpg]

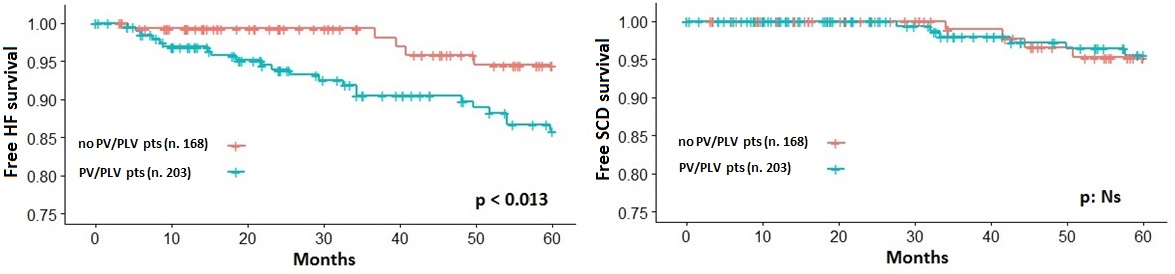

Supplement: Supplementary file 1 [file jcm-09-01636-s001.zip › Genetics Vs CPET in HCM/Figure 3.jpg]
